# A novel framework for expanding temperature intensity-duration-frequency curve utility

**DOI:** 10.1007/s11069-025-07811-1

**Published:** 2025-12-22

**Authors:** Gregory E. Tierney, Megan S. Mallard, Tanya L. Spero, Anna M. Jalowska

**Affiliations:** 1 State Climate Office of North Carolina, North Carolina State University, Raleigh, NC, USA; 2 U.S. Environmental Protection Agency, Research Triangle Park, NC, USA

**Keywords:** Extreme heat, Extreme temperature, Intensity-duration-frequency, Risk assessment, Heat waves, Extreme value analysis

## Abstract

Extreme temperatures—both heat waves and cold snaps—pose a great hazard to human health, property, and infrastructure. Furthermore, projected changes in extreme temperature event frequency and intensity complicates adaptation and mitigation planning for local governments providing cooling/warming centers, entities depending on outdoor labor, and utilities responding to changing energy demand. Flexible tools evaluating the current and future risk of extreme temperature events could help to optimize hazard response. Here, we present an expanded framework for temperature intensity-duration-frequency (TIDF) curve analysis, including an objective fitting algorithm and uncertainty quantification, for the observational record. Prior to this study, the application of TIDF curves has been focused on heat waves. We expand that application to consider the utility of TIDF curves for extreme cold events as well as “near-extreme” events for a more robust quantification of the risk of extreme temperature events. Using a probabilistic approach to extremes, we also calculate confidence intervals, providing additional context for the severity and (where applicable) unprecedented nature of historically extreme events. Further analysis of confidence interval width offers insight to characterize differences in the uncertainty associated with hot and cold extremes. Finally, the flexibility of this new TIDF framework is demonstrated by analyzing two recent extreme temperature events—the 2021 Pacific Northwest heat wave and 2021 Texas cold snap—showcasing the broad utility and potential cross-sector application of a TIDF approach.

## 1 Introduction

Extreme temperature events, such as heat waves and cold snaps, are some of the most ubiquitous and impactful weather hazards around the world. Indeed, the United States National Weather Service recognizes heat as the leading cause of weather-related fatalities in the US (National Weather Service 2023), usually via cardiovascular complications (Desai et al. 2023). These effects can be compounded for outdoor laborers, who can have excessive heat strain with a host of negative health effects (Lucas et al. 2014; Flouris et al. 2018). Heat waves have been correlated with increases in fatal traffic accidents in the US (Wu et al. 2018), intimate partner violence (Sanz-Barbero et al. 2018), and increased suicide rates in the US and Mexico (Burke et al. 2018). Conversely, extreme cold temperatures and cold waves also affect human morbidity and mortality (e.g. Carmona et al. 2016) primarily through pathogenic and infectious disease pathways. These cold events have been shown to be comparable to (Huynen et al. 2001; Kysely et al. 2009; Linares et al. 2015) to perhaps an order of magnitude greater impact in terms of mortality rate (Vardoulakis et al. 2014), especially due to pathogenic and infectious disease related causes affecting the respiratory and cardiovascular systems (see Ryti et al. 2016).

Warming of the Earth’s climate system would lead to either no change or a marked decrease in the intensity and frequency of cold waves, depending on the region of interest (Ceccherini et al. 2016; Van Oldenborgh et al. 2019). However, global increases in temperature have and would continue to simultaneously exacerbate the frequency and effects of heat waves (e.g., Murari and Ghosh 2018; Vicedo-Cabrera et al. 2021; IPCC 2023; Domeisen et al. 2023). This shift in extremes increased temperature-related deaths during heat waves (e.g. Mitchell et al. 2016; Gasparrini et al. 2017; Ballester et al. 2023), spurring adaptation and mitigation efforts across sectors such as public health (Patel et al. 2022; Turek-Hankins et al. 2021), energy (Añel et al. 2017; Abolhassani et al. 2023), and agriculture (Barlow et al. 2015; Gourdji et al. 2013).

Despite numerous past studies on heat events and their impacts, such events still lack a broadly adopted definition. The glossary of the American Meteorological Society begins the definition of a heat wave with “a period of abnormally and uncomfortably hot and usually humid weather…”, a relative definition based on the climatology of the location of interest (American Meteorological Society 2025). Transitioning a relative to an objective definition would need to consider meteorological factors (including local climatology) as well as physiological and societal factors that depend on the characteristics of the impacted population (Robinson 2001). Accordingly, targeted heat wave definitions have proliferated for specific applications with regionally dependent thresholds. For example, Xu et al. (2016) highlighted and compared dozens of definitions from contemporaneous literature only for the impact of heat waves on human mortality. Similarly, meta-analysis performed by Ryti et al. (2016) took a comprehensive approach for “cold spell” definitions by allowing different measures of severity and only restricting the minimum duration.

The variety of definitions for extreme temperature events hinders the development of a conceptual framework able to support multidisciplinary applications. Here, we outline an advantageously flexible approach to extreme temperature event analysis, adapting well-established concepts from extreme value analysis (EVA)—principally Intensity-Duration-Frequency (IDF) curves—for use with extreme temperature events. Rather than relying on predefined non-local thresholds to identify an extreme event, IDF curves return the probabilistic estimated Return Period (RP; or frequency) of an event given its intensity and duration. Initially for hydrological applications (e.g., Sherman 1905; Bernard 1932), IDF curves and their associated return period nomenclature have become ubiquitous tools to communicate extreme event risk, especially when designing infrastructure to withstand heavy precipitation events. (e.g., Jalowska and Spero 2019)

More recently, the IDF curve paradigm has been applied to extreme temperature events through “temperature IDF” (TIDF) or “heat IDF” (HIDF) curves—so named to distinguish them from precipitation IDFs (or PIDFs). TIDFs have been used by Khaliq et al. (2005) to examine heat waves in southern Quebec, and subsequently to examine the recurrence of heat events in Saudi Arabia (Raggad 2018), southern Pakistan (Zahid et al. 2017), China (Li et al. 2021), selected cities in the US (Mazdiyasni et al. 2019) and in South Korea for temperature (Lee et al. 2021). Additionally, TIDFs have been used for wet bulb globe temperature (Shin et al. 2020). These studies have used a range of approaches and distributions to characterize data, including lognormal (Mazdiyasni et al. 2019; Li et al. 2021), generalized logistic (Lee et al. 2021), generalized Pareto (Zahid et al. 2017; Raggad 2018), generalized extreme value (Grady 1992; Mazdiyasni et al. 2019), and loglogistic (Mazdiyasni et al. 2019). This flexible approach can be used for a breadth of applications regardless of geography or metric. In this manuscript, we further evaluate TIDF analysis across hot and cold extremes and propose the framework of a TIDF continuum that can examine “near-extreme” events (e.g., temperatures at the 5th and 95th percentiles) which are often as impactful to human health as the most extreme events. Among the novel aspects of the work presented herein, this TIDF framework has been constructed to objectively select the appropriate statistical distributions and estimate the uncertainty of extreme event characterization. This selection algorithm, including a calculated fit score to automate distribution selection, is accompanied by a detailed discussion of the candidate distribution selection process for temperature applications—which differs from precipitation applications given differences in their statistical characteristics.

The remainder of this manuscript is structured as follows: Sect. 2 outlines the data and methods used to create this expanded framework, with Sect. 3 presenting the results for an example station in Raleigh, North Carolina. The utility of the framework is then explored in Sect. 4, which examines two recent impactful extreme temperature events in disparate regions of the United States, before Sect. 5 offers a summary and thoughts on future expansions and applications of this framework.

## 2 Methods

### 2.1 Data source and preparation

Input observational data are from the Integrated Surface Dataset (ISD) (Smith et al. 2011) distributed by the National Centers for Environmental Information (NCEI). Data for this work focuses on three stations: Raleigh-Durham International Airport in North Carolina (KRDU), Seattle-Tacoma International Airport in Washington (KSEA), and the Dallas/Fort Worth International Airport in Texas (KDFW). The ISD is a database compiling station observations at the hourly and three-hourly (synoptic) frequency from over 35,000 global stations. Where possible, it contains observational records back to 1901, merging them into a single unified dataset for scientific use. Given that the ISD is not a reanalysis product but rather an observational database, its continuity is dependent on the continuity of the station data compiled. Additionally, as there may be human observational/input errors, the ISD has quality control processes, outlined by Lott (2004), to flag such errors. Hourly temperature observations from the ISD are available for the calendar year; however, this partitioning potentially interrupts extreme temperature events during the Northern Hemisphere winter and Southern Hemisphere summer. To maximize temporal and geographical flexibility of our framework, each 12-month period follows the water year (1 October–30 September in the Northern Hemisphere); all time periods for TIDFs within this manuscript therefore begin on 1 October of the first listed year, and end on 30 September of the last listed year.

All baseline analyses within this manuscript are based on 30 water years of observations spanning 1 October 1990 to 30 September 2020, aligned with the World Meteorological Organization (WMO) recommendation for a climatological period (WMO 2015). TIDF curve analyses based on shorter data records (~ 15 years or less) tend to be quite unstable as intensity (i.e., severity) at all return periods becomes highly sensitive to introducing (or withholding) additional data. Furthermore, the confidence intervals around TIDF curves generated from such short data records are large enough to render such curves functionally impractical. Therefore, data records of 30 years or longer are used throughout this analysis to achieve robust TIDF curves that minimize the effects of background non-stationarity to baseline temperatures, an issue with longer periods of record. This is a common issue across many climate applications, including drought assessment (see Lisonbee et al. 2024). Procedures to mitigate such issues range in computational expense and complexity, however, even more straightforward methods such as a 30-year moving window, suggested by Hoylman et al. (2022) for drought applications, should similarly address this issue for TIDF curves. The impact of the length of the data record on TIDF values and confidence intervals is explored further in Sect. 4.

### 2.2 Annual percentile series and distribution selection

TIDF calculation begins by creating Annual Maximum Series (AMaxS) for extreme heat and Annual Minimum Series (AMinS) for extreme cold at event durations of interest. For this analysis, 13 event durations are considered: three sub-daily durations (2, 6, and 12 h) and ten daily durations from 24 to 240 h (1 to 10 days) stepped every 24 h (1 day). The AMaxS is created by evaluating the average temperature of every possible candidate event of a given duration during the year (a “new” candidate event is initialized every hour) and selecting the candidate event with the highest average temperature. Similarly, the AMinS is created by selecting the candidate event with the lowest average temperature. The TIDF method is expanded to incorporate near-extreme events by creating a generalized Annual Percentile Series (APS) and selecting the candidate event at the 5th (cold) or 95th (hot) percentiles. Within the APS framework, the AMaxS nominally corresponds to the “100th percentile” and the AMinS to the “0th percentile”, or the absolute ranges of the observational data record at each duration for a given location. Initializing candidate events every hour can allow a single extreme observation (e.g., a single-day event) to influence several durations (e.g., overlapping multi-day average temperatures). Accordingly, TIDF graphs are generated for near-extreme percentiles, such as the 5th or 95th percentile from the full data record. This method of candidate event initialization avoids arbitrary choices around “separation time” between events or calculating averages in disparate time blocks, which would consequently underestimate extremes. Overall, using a rolling average with hourly candidate event initialization maximizes flexibility for numerous TIDF applications.

Calculating the APS produces a dataset of temperatures representing the number of years in the underlying data record. The APS is then subsequently fit via an objective algorithm to determine a characteristic distribution for the station at that percentile. To identify plausible characteristic distributions for TIDF applications, all available distributions for continuous variables were evaluated using the SciPy statistical functions module, version 1.14.0 (Virtanen et al. 2020). This initial list of over 100 distributions (available in scipy.stats) underwent an initial suitability assessment based on foundational assumptions and the results of exploratory analysis, specifically that the underlying distribution that will best fit the APS is (1) approximately symmetric and (2) has both left and right tails. These assumptions apply to temperature applications and are inappropriate in choosing distributions for precipitation IDF curves (PIDFs).

Screening to satisfy these two assumptions identified 29 potential distributions that were evaluated at several sites, where goodness-of-fit from the maximum likelihood estimation method was tested using the Kolmogorov–Smirnov (K–S) method (Massey 1951). Although distributions with three or more parameters (such as the generalized normal and generalized logistic distributions) achieved marginally improved quantitative fit scores, over-fitting often led to non-physical solutions, such as arbitrary increases and decreases in intensity for some frequencies at longer event durations. Furthermore, distributions utilized for PIDFs, such as the generalized extreme value (GEV) distribution, generate non-physical tails for record extremes—demonstrating some unique considerations for creating TIDFs versus PIDFs. Physically plausible solutions are required, so two-parameter distributions that performed the best in our exploratory analysis are selected. This resulted in a candidate list of four distributions: cosine, hyperbolic secant, logistic, and normal. The uniform distribution is added as a data quality flag, as this distribution is unlikely for error-free datasets. Crucially, these four distributions perform well regardless of the percentile evaluated, creating a consistent algorithm across the spectrum of extreme and near-extreme events.

An algorithm was developed to objectively select the appropriate distribution for each unique combination of station, data record, and percentile. After this combination is defined, maximum likelihood estimation is used to fit the corresponding APS for each of the 13 event durations (from 2 to 240 h, as described earlier) using each of the five candidate distributions. A fit score is then calculated based on the K–S test statistics for each distribution: the mean of the 13 K–S test scores (one from the APS at each of the 13 event durations) multiplied by the standard deviation of the 13 K–S test scores, where smaller fit scores indicate better fits. This construction balances a high-quality fit with a consistency of fit across all event durations. The distribution with the lowest “fit score” is chosen for that combination of station, data record, and percentile. We further constrain the algorithm such that the mean p-value of the chosen distribution must be greater than or equal to 0.4 across all provisional fits in order to screen out poorly fitted annual series, although this condition is rarely triggered. The construction of this algorithm promotes objective adaptability, transparency, and independence in the analysis of each data record.

After the final fit is determined for the APS, the quantile function is evaluated for the resulting fit at RPs of interest. For brevity, temperature thresholds at three RPs are highlighted in this study: common events (1-in-2-year event; 50% annual exceedance probability), rare events (1-in-10-year event; 10% annual exceedance probability), and potentially record-breaking events (1-in-100-year; 1% annual exceedance probability).

### 2.3 Constructing confidence intervals

The average RP for a 1-in-100-year event is substantially longer than the 30-year reference period, the length recommended by the WMO (2015). Accordingly, the 1-in-100-year and 1-in-50-year RPs approach or exceed the maximum length of the data record at many stations, motivating communication of the uncertainty in temperature thresholds, particularly at longer RPs. Indeed, this uncertainty also exists for events with RPs shorter than the data record. Therefore, complete risk assessment of extreme temperature events using TIDF curves includes calculation and communication of the confidence interval (CI) around the threshold intensity for all event durations and RPs.

Like the approach used to apply one of the five candidate statistical distributions to the data record, the CI calculation is also designed to be independent of the data distribution, rather than employing a mix of analytical (for normal and logistic distributions) and empirical methods. Therefore, a bootstrapping method is applied to retain flexibility, which can accommodate additional candidate distributions or different distributions for different event durations, left for future work. Here, the 90% CI is shown, although the framework is flexible enough to accommodate other CIs.

The calculation of CIs is unique for each event duration and begins with resampling the APS (with replacement) to obtain a “new” dataset equal in size to the original APS. The resampled APS is fit with the original statistical distribution chosen by the algorithm, with the two parameters of the best fit distribution for the resampled APS recorded as P1 and P2. This resampling and refitting process is repeated 7500 times, resulting in 7500 (P1, P2) pairs. Deriving a resampled APS 7500 times is somewhat arbitrary; but it sufficiently exceeds the generally accepted minimum of ~ 1000 resampled datasets with minimal additional computing expense. After resampling, the 5th and 95th percentile of the 7500 P1 and P2 values are calculated (noted here as P1_5_, P1_95_, P2_5_, and P2_95_). These parameter percentile values for P1 and P2 are combined to create parameter pairs for four “extreme” distributions—of the same type as the distribution originally selected by the algorithm—with constructions (P1_5_, P2_5_), (P1_5_, P2_95_), (P1_95_, P2_5_), and (P1_95_, P2_95_). Like the fitting of the original observed APS, the percentage point function is then evaluated at the RPs of interest for each of these four “extreme” distributions, resulting in four temperatures for each RP (one from each “extreme” distribution). The 90% CI is then defined by retaining the minimum and maximum temperatures of the four extreme distributions as the lower and upper bounds of the CI, allowing TIDFs to be accompanied by estimates of the uncertainty in the intensity of extreme events.

## 3 Results

**Fig. 1:**
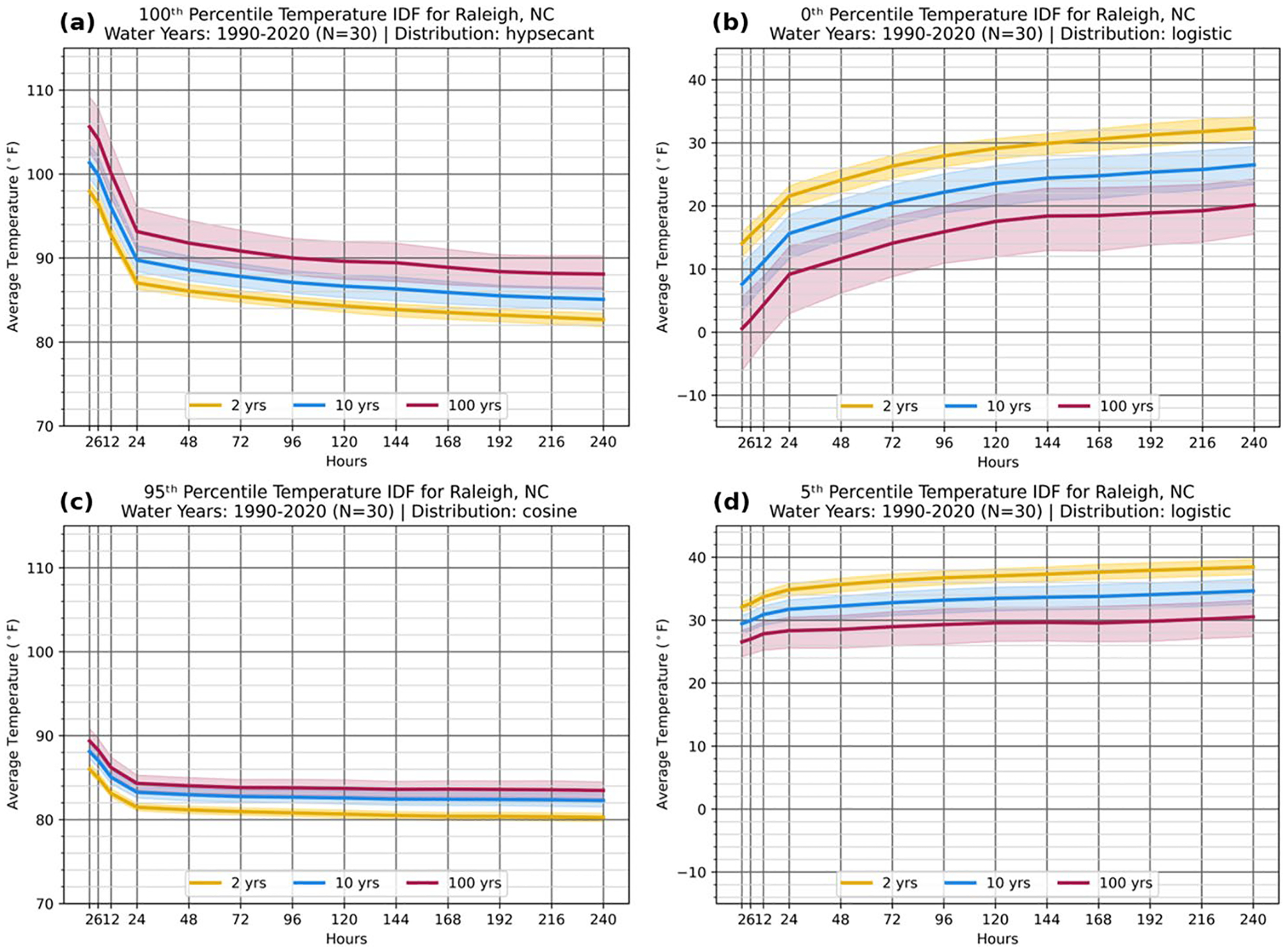
TIDF curves (based on 1990–2020 observational record) for Raleigh-Durham, NC (KRDU) at the **a** nominal 100th percentile, **b** nominal 0th percentile, **c** 95th percentile, and **d** 5th percentile for events at return periods of 1-in-2-years (yellow), 1-in-10-years (blue), and 1-in-100-years (red). Shading indicates the 90% confidence interval. Titles over each panel give the percentiles used, years considered, and information on the distribution utilized

The new TIDF framework is demonstrated using the observations from the Raleigh-Durham International Airport in North Carolina (KRDU); however, the analysis could be performed with any high-quality observed *or* modeled data series. KRDU is an active site with an observational history at its current location initiating on May 18, 1944. 30 water years beginning 1 October 1990 and ending 30 September 2020 are used. Figure 1 presents four TIDFs for KRDU corresponding to the 0th, 5th, 95th, and 100th percentiles. Temperature is primarily shown and analyzed in degrees Fahrenheit to correspond to units most familiar to the US public.

Figure 1a displays the expected value of “100th percentile” (AMaxS) events for RPs of 1-in-2-year, 1-in-10-year, and 1-in-100-year events. As an example of how to interpret these plots, the expected average temperature for a 1-in-100-year, 4-day event for KRDU is 90.0 °F (32.2 °C). Alternatively, using the 90% CI bounds, this event threshold is between 88.0 °F and 92.3 °F (31.1 °C and 33.5 °C). A qualitative check of the TIDF results can be performed by considering the all-time high temperature at a station against an extreme event as calculated by the TIDF framework, here represented by the 1-in-100-year value for an event duration of 2 h. These two values should be comparable, especially if the record was established during the TIDF analysis period. For KRDU, the record maximum observed temperature is 106.0 °F (41.1 °C; observed on 5 July 2024, after the climatological data record included), which compares well to the TIDF 1-in-100-year, 2-h event of 105.4 °F (40.8 °C). The objective selection algorithm for the best-fit distribution results in monotonic TIDF curves, with an abrupt slope change between sub-daily event durations of 2, 6, and 12 h and event durations of 24 h or longer, which incorporate overnight low temperatures.

TIDFs are typically used for extreme maximum temperature events (the nominal 100th percentile), as in Fig. 1a, but they are analyzed here for cold extremes (Fig. 1b) and both hot and cold near-extreme (Fig. 1c, d) temperature events. The first of these novel applications is at the nominal “0th percentile” TIDF, based on the AMinS. Extreme cold TIDFs generally have smoother transitions between the 12- and 24-h event durations and wider confidence intervals than other percentiles—explored in detail later.

Additionally, this TIDF framework uses the full APS by examining “near-extreme” events (here, the 95th and 5th percentiles) that can be as impactful as the record extremes and offer a more robust view of high-impact events by showing the thresholds for the hottest and coldest 5% of averaging windows at each duration. Using these intermediate percentiles necessitates a closer examination of how event duration is considered and increased discretion in the interpreting the resulting analysis. To derive the APS, all possible events in the year of the event duration of interest are considered, with a new event beginning each hour. Thus, there are 8760 (or 8784 in leap years) possible overlapping events in each year. This method provides the most extreme estimate for the APS but avoids arbitrarily defining the overlap between possible events. It also provides a consistent estimation method regardless of percentile or event duration. At the 24-h duration, these plots correspond to approximately the 18th hottest day (Fig. 1c) and 18th coldest day (Fig. 1d) of the year. At KRDU, these correspond to 24-h average temperatures of 84.3 °F (29.1 °C; 95th percentile) and 29.9 °F (− 1.2 °C; 5th percentile) for 1-in-100-year events. These near-extreme values can inform stakeholders by providing additional context around the severity and rarity of extreme temperature events, and this framework can be used to examine other less-extreme representations of risk via RPs. TIDFs across a range of percentiles can improve resource management across sectors where response to both extreme (e.g., 0th and 100th percentile events) and near-extreme (e.g., 5th and 95th percentile) events is important to consider when protecting human and ecosystem health, including mitigation and adaptation measures in urban areas (e.g., heating/cooling shelters), agriculture, and ecological impacts.

**Fig. 2:**
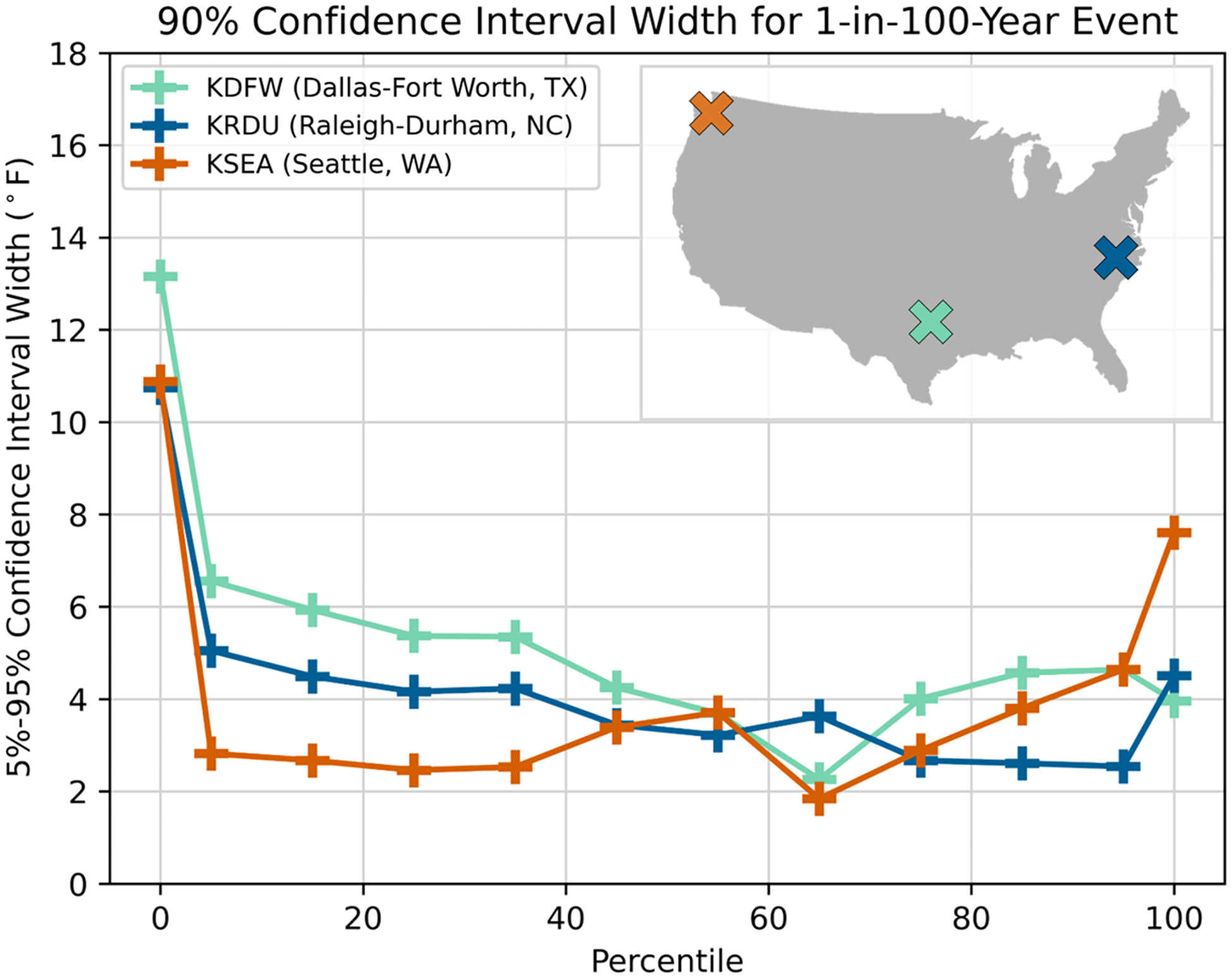
Average 90% CI width (in °F) for a 1-in-100-year event (averaged across all event durations) versus event percentile for KDFW (teal), KRDU (dark blue), and KSEA (orange). Data are provided at the 0th and 100th percentile, as well as in increments of 10 from the 5th to 95th percentile. The location of each station is shown on the map in the upper right

The uncertainty around these hot and cold thresholds (90% CIs in Fig. 1) reflects the station and percentile analyzed. TIDFs for hot extremes generally exhibit smaller CI widths than TIDFs for cold extremes (Fig. 1a, b), illustrating lower confidence in the range for the cold extremes. Figure 2 presents the relationship between CI width, event percentiles, and station location for the three stations used in this manuscript: KRDU, Seattle, Washington (KSEA), and Dallas-Fort Worth, Texas (KDFW), evaluating the width of the 90% CI for 1-in-100-year events at the 0th and 100th percentiles, as well as every 10th percentile from the 5th to 95th percentile.

Although these three stations are in different climate regimes in the US, similar patterns in CI width are observed across all percentiles. At each location, the extremes (nominal 0th and 100th percentiles) have the widest CIs, which is noted here and left for future study. A contrast between the uncertainty around cold extremes versus warm extremes is also apparent, with the maximum uncertainty for all three sites found around extreme cold events. Other than for the absolute extremes, uncertainty is generally constrained to 2° to 6 °F (1.1 °C to 3.3 °C), regardless of station, percentile, or distribution utilized to fit the data.

## 4 Contextualizing recent extreme events with TIDFs

Two record-breaking temperature events are analyzed using TIDFs developed using the antecedent historical record, illustrating the value added from a TIDF analysis, and how that analysis is affected by subsequently including those events into the input data record.

### 4.1 2021 Pacific Northwest heat wave

In late June 2021, the extreme heat in the US Pacific Northwest and southwestern Canada set several high temperature records, including a new Canadian record high temperature of 121 °F (49.6 °C) in Lytton, British Columbia and high temperature records in several other cities across the region. This event has led to studies on its impact (e.g., White et al. 2023; Henderson et al. 2021; Nori-Sarma et al. 2022), forecast perspectives (e.g., Emerton et al. 2022; Lin et al. 2022), and contributing meteorological factors (e.g., Overland 2021; Loikith and Kalashnikov 2023; Lin et al. 2022). Fleishman et al. (2025) contains a synthesis of publications on the event. Here, we characterize the rarity of this event from a TIDF perspective.

**Fig. 3:**
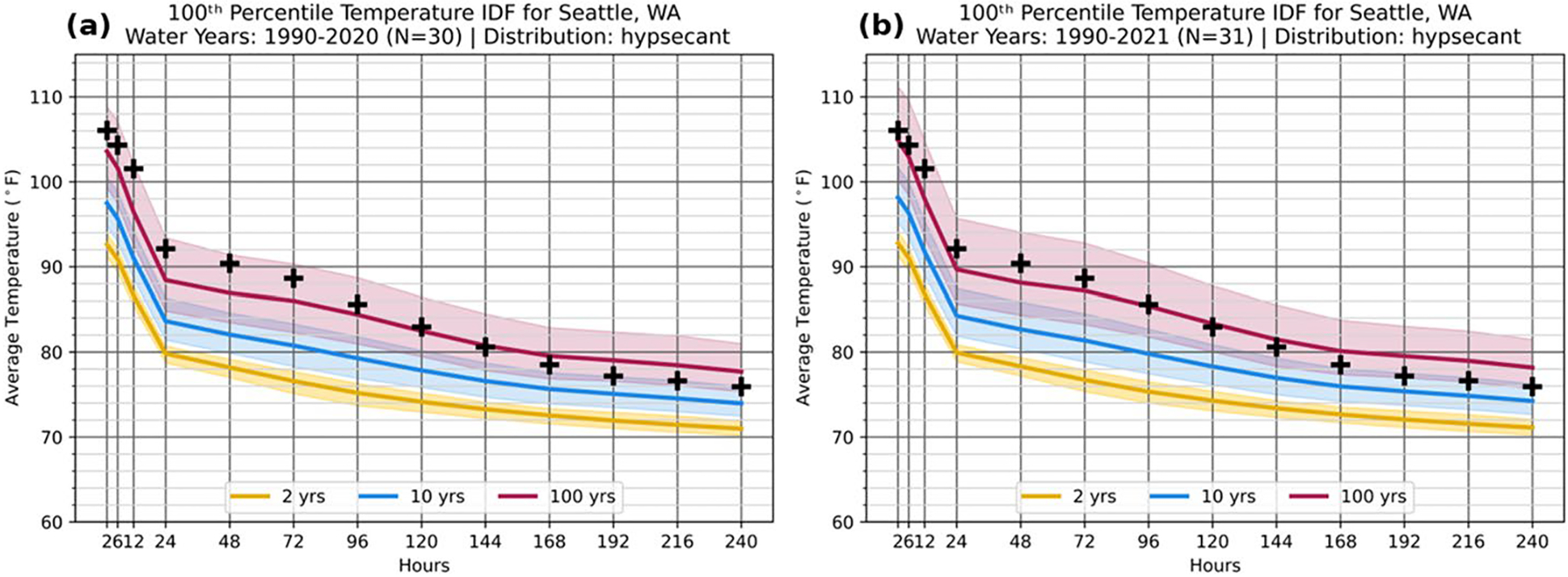
TIDF curves at the nominal 100th percentile for Seattle, Washington (KSEA), for events at return periods of 1-in-2-years (yellow), 1-in-10-years (blue), and 1-in-100-years (red) based on the observational record from water years spanning **a** 1990–2020 (antecedent observational record) and **b** 1990–2021 (including the record-breaking event in the observational record). The annual maximum series for KSEA during the 2020–2021 water year is superimposed in black crosses identically on each panel

Observations from the Seattle-Tacoma International airport (KSEA) are used to characterize the event. This analysis uses the 30-year period immediately prior to this event (water years 1990–2020), with Fig. 3a presenting the KSEA TIDF curves and CIs at the nominal 100th percentile (extreme heat) for 1-in-2-year, 1-in-10-year, and 1-in-100-year RPs. Overlaid on these curves are a series of markers representing the hottest temperature at KSEA for each event duration during the 2020–2021 water year (1 October 2020–30 September 2021). Given the extreme nature of the June 2021 event relative to the rest of the year, the maxima for the year can serve as a proxy for the June 2021 event. Most notably in Fig. 3a, these maxima are warmer than the 1-in-100-year TIDF curve out to event durations of 120 h (5 days) but remain within the CI of a 1-in-100-year event. At event durations of 48 h or shorter, the 2021 maxima exceed the 1-in-100-year threshold by more than 4 °F (2.2 °C) but still fall within the CI of the 1-in-100-year event. By including CI in this TIDF analysis, a reasonable range of possible temperatures is given for such an event, rather than a specific thresholding for extremes. The TIDF analysis was extended to longer RPs beyond 1-in-100-year events to quantify an approximate median rarity for the June 2021 event. This event is estimated as a 1-in-500-year event (Online Resource 1), broadly consistent with other independent contemporary analyses (e.g., Philip et al. 2022; Bartusek et al. 2022; McKinnon and Simpson 2022; Overland 2021).

Expanding the input data record length for TIDFs to incorporate newer data can illustrate the impact of a record-breaking event on TIDFs. For the 2021 Pacific Northwest heat wave at KSEA, this impact is demonstrated by comparing the “pre-event” TIDF (1990–2020; Fig. 3a) to TIDFs that include the event (1990–2021; Fig. 3b). As expected, including the 2021 extremes increases the median thresholds for all RPs; however, even within the post-event TIDF, the 2021 event still exceeds a 1-in-100-year event at short durations. Note that the magnitude of the 2021 event’s effect on KSEA TIDFs is not uniform, but instead reflects where the event most exceeded the existing TIDF curves, with the greatest increases at sub-daily durations and longest RPs. Notably, the 1-in-100-year median event increases by an average of 0.95 °F (0.53 °C) across all durations, with a maximum increase of 1.5 °F (0.83 °C) at 12-hr. In comparison, the 1-in-10-year median event increases by 0.49 °F (0.27 °C) on average, with a maximum increase of 0.74 °F (0.41 °C) at 12-h. The 1-in-2-year median event increases by an average of only 0.13 °F (0.07 °C), with a maximum increase of 0.16 °F (0.09 °C) at 2-hr. Similarly, the unprecedented nature of the event affects the 90% CIs around the event medians in the KSEA 100th percentile TIDF, particularly in the longest RPs, as on average, the 1-in-100-year event CI increases in width by 13%, from 7.6 °F (4.2 °C) to 8.6 °F (4.8 °C). In contrast, the average CIs for 1-in-2-year events increase slightly from 2.20 °F (1.33 °C) to 2.24 °F (1.36 °C).

### 4.2 2021 Texas cold snap

Although heat is the leading weather-related cause of death, cold extremes can be similarly devastating for life, agriculture, and infrastructure, as demonstrated by the cold snap that enveloped most of North America in early to mid-February 2021. Stretching southward from Canada to northern Mexico, this event set daily record low temperatures in cities such as Dallas, Texas (− 2 °F/− 18.9 °C on February 16), and Houston, Texas (13 °F/− 10.6 °C on February 16), part of a total of 2311 daily minimum temperature records set in the event, as well as 66 all-time minimum temperature records (Bolinger et al. 2022). Media coverage of this event centered on Texas, where the extremely cold temperatures led to an infrastructure crisis, including cascading issues with the ERCOT power grid (Busby et al. 2021; Shrestha et al. 2023). The TIDF framework can also be used to contextualize the severity of cold snaps. The extreme nature of the conditions that led to this crisis can be evaluated using the 0th percentile TIDFs for the region, as demonstrated using observations from the Dallas/Fort Worth International Airport, Texas (KDFW).

**Fig. 4:**
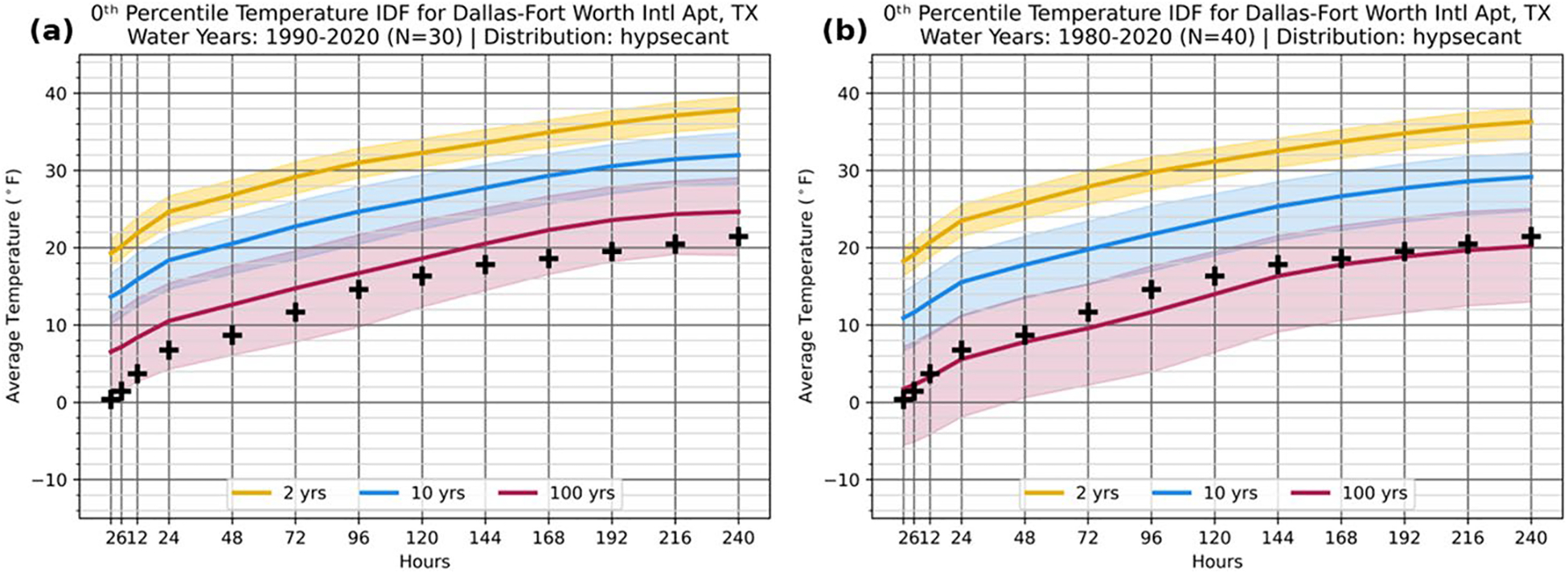
TIDF curves at the nominal 0th percentile for Dallas-Fort Worth, Texas (KDFW) for events at return periods of 1-in-2-years (yellow), 1-in-10-years (blue), and 1-in-100-years (red) based on the observational record from water years spanning **a** 1990–2020 and **b** 1980–2020. The annual minimum series for KDFW during the 2020–2021 water year is superimposed in black crosses identically on each panel

Analogous to Fig. 3a for the Pacific Northwest heat wave, Fig. 4a consists of the “pre-event” (1990–2020) 0th percentile TIDFs for KDFW. The 2020–2021 water year minima exceed the 1-in-100-year threshold at all durations from 2 h to 10 days, with the greatest exceedance found at sub-daily durations and at 192 and 216 h (8 and 9 days), with the latter period emphasizing the prolonged impact of this event. The record-breaking impact of this event across a range of durations can be concisely communicated within the IDF framework, rather than focusing on records covering only one duration, such as daily records. Expanding the analysis to include longer RPs (Online Resource 2) indicates that, relative to the 1990–2020 baseline, the 2021 event is estimated to be a 1-in-750-year event at the shortest event durations and a 1-in-100-year to 1-in-500-year event for most other event durations. However, the event also falls primarily within the 90% CI of a 1-in-100-year event, illustrating the importance of including CIs in extreme event analysis to account for uncertainty in the data.

In this case, using 1990–2020 as a baseline omits record cold outbreaks in February and December 1989, among others. Just as the Pacific Northwest heat wave example illustrates how record-breaking events can modify TIDFs, extending a baseline back in time can have similar effects. To that end, Fig. 4b demonstrates the effect of lengthening the data record to include these past events into the TIDF analysis for KDFW, presenting the 0th percentile IDF for KDFW based on a 1980–2020 baseline (40 water years). When adding water years 1980–1990, the event still falls within the 1-in-100-year CI but would be considered a 1-in-50-year to 1-in-100-year event at median thresholds. Regardless of the climatological baseline, this estimate is comparable to analysis by Doss-Gollin et al. (2021) who used three datasets with varying baseline periods (each with a minimum period of 60 years). This difference in characterizing rarity with RPs further underscores the advantages of including CIs in TIDF analysis to quantify uncertainty in risk analysis. Including CIs produces consistent bounds around the RPs of such extreme events, even as specific estimates may change with the method or data used.

## 5 Conclusions

Both heat waves and cold snaps can significantly impact human health, infrastructure, and many aspects of society. Using specific thresholds to define extreme temperature events across broad geographical regions leads to a wide array of definitions, owing to myriad climatological regimes potentially present within a geographical region. These definitions can vary in magnitude, event duration, and metric, and while they may perform well in specific use cases, they are often not well-suited to a wide variety of applications. This work introduces a different approach—a flexible paradigm for evaluating and contextualizing extreme temperature events using TIDF curves with CIs. This approach contextualizes hot *and* cold events using a consistent methodology and without requiring specific thresholds or durations (such as 90 °F or 32 °F) that may be less appropriate for certain applications or locations.

While most previous work around TIDF curves has focused on using annual maximum series to analyze the hottest extremes, this work generalizes that concept by using annual percentile series (APS) to develop TIDF curves. This framework facilitates the analysis and contextualization of extreme events for both hot and cold extremes at individual locations using the same overarching method that adapts to the data record. Uniquely, this framework also permits the analysis of near-extreme TIDFs, such as the 5th and 95th percentiles, that can be as impactful as record-breaking extreme events. Expanding TIDF analysis across a spectrum of events provides flexibility at individual locations using a concise data-based method that is easily understood yet remains flexible for specific planning needs, from adaptation measures for an upcoming multi-day cold snap to planning for adequate cooling shelters during a near-record heat wave.

Furthermore, the framework introduced here uses an objective algorithm to select the best distributional fit for the APS from five pre-screened statistical distributions, including one quality control flag distribution. Using an objective algorithm avoids inherent biases possible with human-selected statistical distributions. Transitioning from subjective distribution selection (the predominant method used in TIDF literature) to an objective algorithm (presented here) expedites automation of the framework, making TIDF tools and analysis available and accessible to more stakeholders. This objective algorithm also includes the option to calculate and display CIs based on bootstrapping, which can adapt to any distribution. These CIs offer insight into the envelope of potential event possibilities at a given location, even when an event of such magnitude is not yet present in the observational record. As probabilistic approaches for risk assessment and hazard communication are popularized, TIDF tools featuring CIs give stakeholders a customized understanding of the present and future risks of extreme temperature events based on the available data at their location, potentially aiding more efficient allocation of resources to protect their communities.

## Supplementary Material

Supplement1

**Supplementary Information** The online version contains supplementary material available at https://doi.org/10.1007/s11069-025-07811-1.

## Data Availability

Datasets in this research are included in NOAA National Centers for Environmental Information (2001), which is openly available at the locations cited in the reference section.
