# Supplementary material for: A novel framework for expanding temperature intensity-duration-frequency curve utility
Source: Nat Hazards (Dordr). Author manuscript; Available in PMC 2026 May 5. (PMC13138118; doi:10.1007/s11069-025-07811-1)
Supplement: Supplement1 [file NIHMS2132775-supplement-Supplement1.docx]

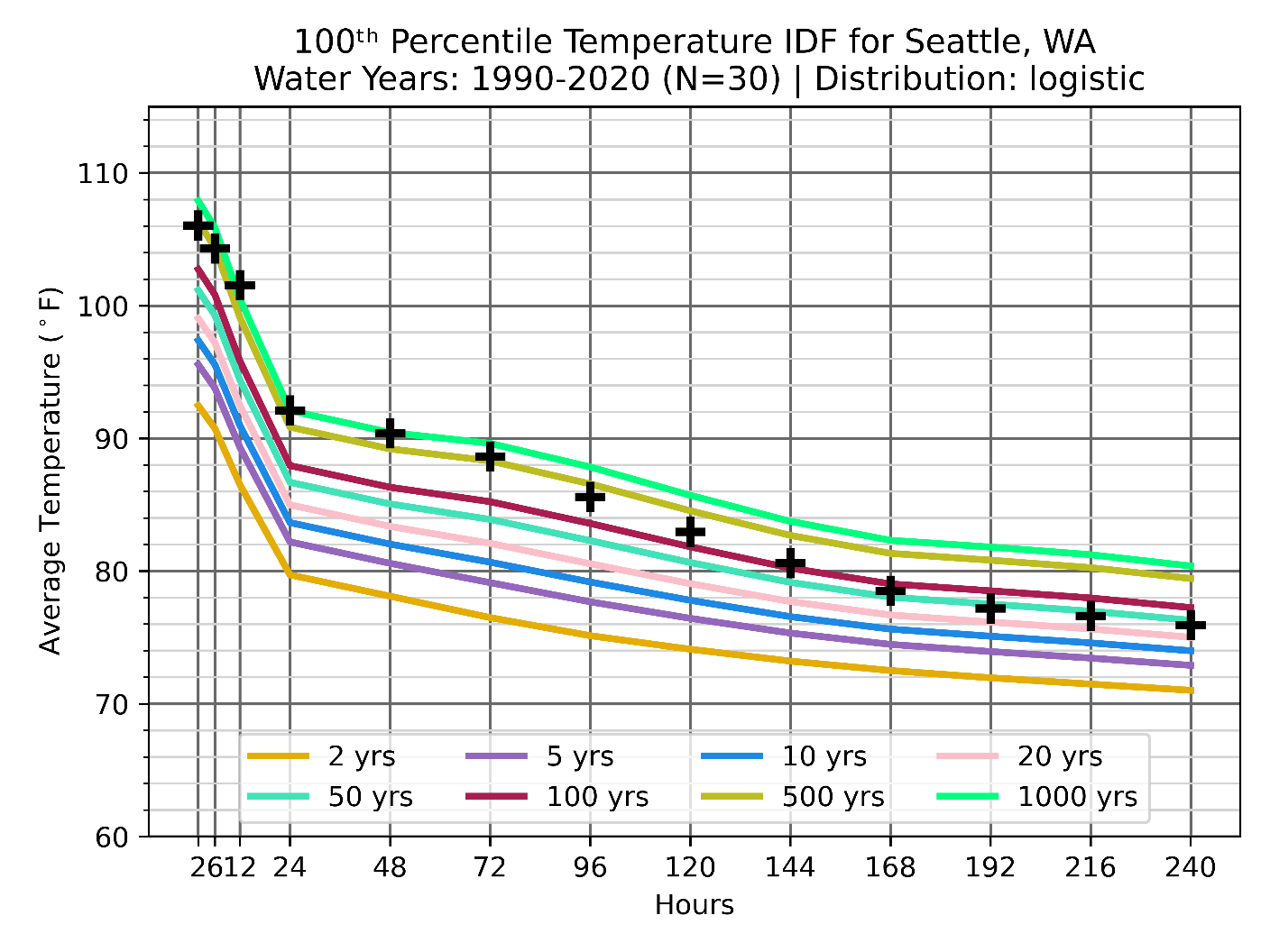


**Online Resource 1** TIDF curves at the nominal 100^th^ percentile for Seattle, WA (KSEA) for events at return periods ranging from 1-in-2-year to 1-in-1000-year event based on the 1990-2020 observational record. The annual maximum series for 2021 at KSEA is superimposed in black crosses.


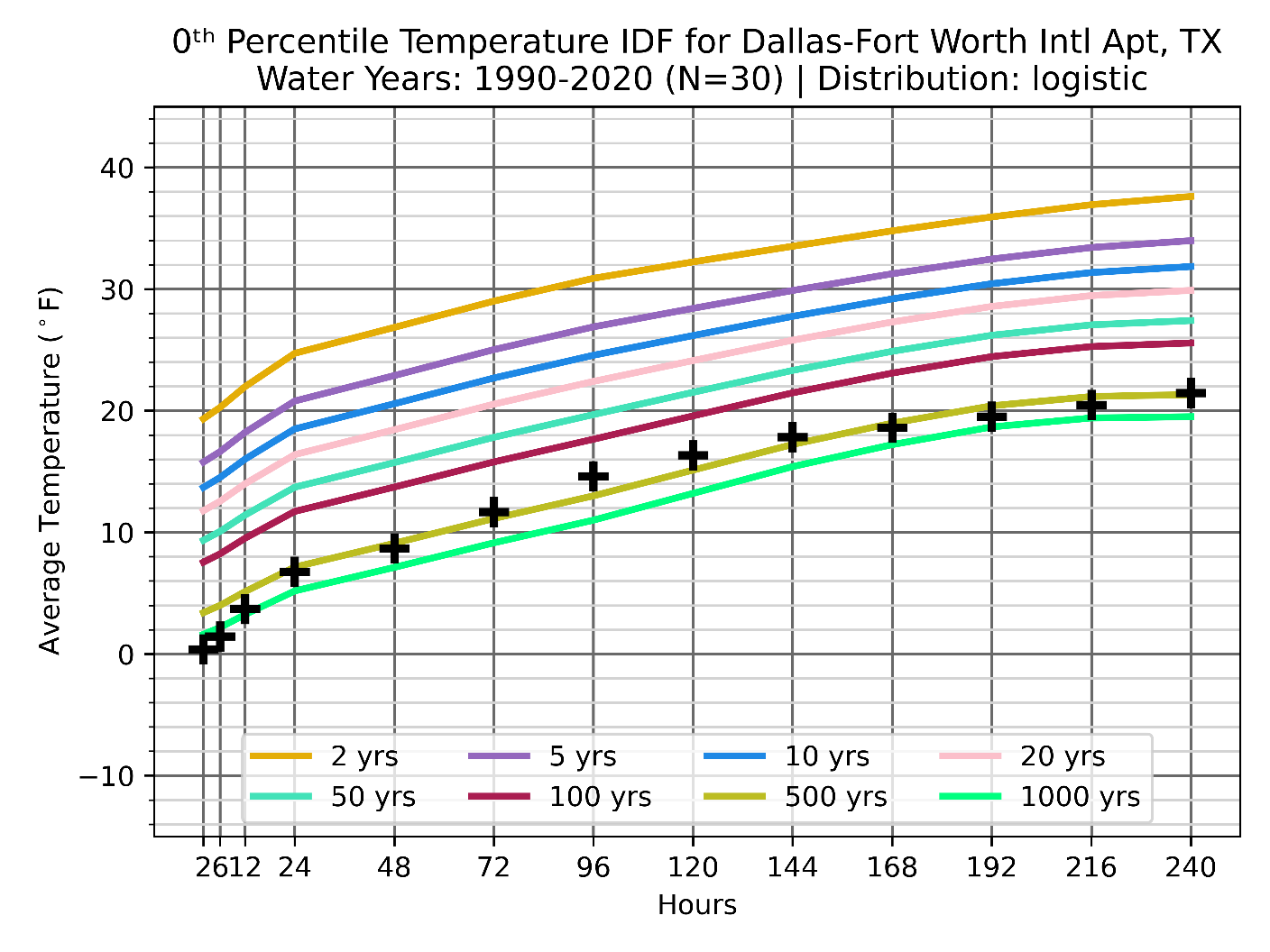


**Online Resource 2** TIDF curves at the nominal 0^th^ percentile for Dallas, TX (KDFW) for events at return periods ranging from 1-in-2-year to 1-in-1000-year event based on the 1990-2020 observational record. The annual minimum series for 2021 at KDFW is superimposed in black crosses.
